# Epstein-Barr virus-associated autoimmune hemolytic anemia: a clinical report and review of literature

**DOI:** 10.1186/s13052-025-01966-0

**Published:** 2025-04-17

**Authors:** Salvatore Accomando, Simona Scalzo, Giulia Angela Restivo, Carlo Provenzani, Giovanni Corsello, Mario Giuffrè

**Affiliations:** 1 Department of Health Promotion, Mother and Child Care, Internal Medicine and Medical Specialties “G. D’Alessandro”, University of Palermo, Palermo, Italy; 2 Emergency Department, ARNAS Ospedali Civico, Di Cristina e Benfratelli, Palermo, Italy; 3 Pediatric Hematology and Oncology Unit, ARNAS Ospedali Civico, Di Cristina e Benfratelli Palermo, Palermo, Italy

**Keywords:** Epstein-Barr virus (EBV), Anemia, Children, Literature review

## Abstract

**Background:**

Epstein-Barr virus (EBV) infection is a common disease both in children and adults, but can lead to several complications; involvement of the blood system is often described, particularly neutropenia and thrombocytopenia, but autoimmune hemolytic anemia is rarely seen.

**Case presentation:**

A 12-year-old female was admitted to the “G. Di Cristina” Children’s Hospital of Palermo for jaundice and dark urine. Laboratory investigations revealed anemia, increased levels of total and undirect bilirubin, and elevated transaminases, serum lactate dehydrogenase, and reticulocyte count; a peripheral blood smear showed anisocytosis, and the direct antiglobulin test (DAT) for cold agglutinins was positive. The laboratory evaluation of infectious disease showed the presence of EBV VCA IgM and IgG. A diagnosis of acute autoimmune hemolytic anemia EBV related was made: the patient was initially treated with intravenous methylprednisolone and then with intravenous immunoglobulin, which led to a progressive clinical improvement until complete remission.

**Conclusions:**

Autoimmune hemolytic anemia is rarely associated with EBV infection; a review of the English literature revealed only 16 cases. Patients with autoimmune hemolytic anemia should always be evaluated for EBV serology, even in the absence of the typical clinical and hematological features of infectious mononucleosis. For these patients, good prognosis is generally expected.

## Background

Epstein-Barr virus (EBV) is responsible for the infectious mononucleosis clinical syndrome, which mainly consists by fever, malaise, headache, lymphadenitis, and pharyngitis [1]; it is usually a self-limiting disease, occurring in childhood, and resolves spontaneously in a few weeks. EBV infection can lead to several complications: the most common are enlarged spleen and liver, with elevated transaminases, splenic rupture, acute cholecystitis, and airway obstruction. Other rare complications are myocarditis, encephalitis, hemophagocytic lymphohistiocytosis, and pancreatitis [1]. Occasionally, it is described an involvement of the hematologic system, which is characterized by neutropenia, thrombocytopenia, and, rarely, autoimmune hemolytic anemia [1, 2],. Here we report on a 12-year-old girl with hemolytic anemia secondary to cold agglutinins due to EBV infection; a review about this topic was also conducted thereafter.

## Case presentation

A 12-year-old female was admitted to the “G. Di Cristina” Children’s Hospital of Palermo for jaundice and dark urine. She was subfebrile and other vital signs were normal. Physical examination was notable for jaundice, no rashes, diarrhea, vomiting, arthralgias, dysuria or other symptoms were complained. Laboratory investigations showed hemoglobin 8,7 g/dL, platelets 180.000/mm3, white blood cells count 17.900/mm3 (neutrophils 23%, monocytes 7%, and lymphocytes 68%), total bilirubin 7,08 mg/dL (normal range 0–1.00), undirect bilirubin 5,23 mg/dL (normal range 0-0.29), aspartate aminotransferase 122 U/L (normal range 0–32), alanine aminotransferase 101 U/L (normal range 5–33), lactate dehydrogenase (LDH) level 1303 U/L (normal range 135–214). She was hospitalized for suspected hemolytic anemia. There was no history of illness or recent use of drugs except for steroids and ibuprofen; no prior history or family history of hemolytic anemia. Immunoglobulins were normal and no autoantibodies were found. By the next day, her hemoglobin dropped to 6,3 g/dL, reticulocyte count was 4.57% (normal range 0.50–2.50%) and peripheral blood smear showed abnormal red blood cell morphology with anisocytosis. Serological tests for mumps, parotitis, rubella, EBV, cytomegalovirus, varicella-zoster virus, herpes simplex virus, coxsackie virus, and mycoplasma were all negative, except for the presence of EBV VCA IgM and IgG. The direct antiglobulin test (DAT) for cold agglutinins was positive for C3 3+. Thus, a diagnosis of EBV-related acute autoimmune hemolytic anemia was made. Exudative pharyngotonsillitis and cervical lymphadenopathy were not present, except for a mild hepatosplenomegaly. Firstly, therapy with intravenous methylprednisolone (2 mg/kg/day) was started and, after two days, intravenous immunoglobulin (0,8 mg/kg) therapy was added. Few days later, she improved clinically: hemoglobin increased to 8,5 g/dL, reticulocyte count was 16,8% and, LDH and bilirubin levels gradually decreased. She was discharged home on the eight days of hospitalization. At home, oral methylprednisolone was gradually tapered.

## Discussion and conclusions

Autoimmune hemolytic anemia (AIHA) is a rare manifestation of EBV infection: it is characterized by the hemolysis of red blood cells caused by autoantibodies directed against erythrocyte surface antigens. There are two main types of AIHA: (a) warm AIHA, caused by increased extravascular erythrocyte destruction by immunoglobulin G (IgG) autoantibodies, with or without complement activation, at normal body temperature; (b) cold AIHA, caused by immunoglobulin M (IgM) autoantibodies, that bind erythrocytes at temperatures below 37 °C, resulting in complement activation and predominantly intravascular hemolysis. During EBV infection, cold agglutinins may develop in up to 60% of patients, but only 0.5-3% of them, because of high titer of auto-antibodies, evolve into clinically significant hemolysis [3]. The pathogenic mechanism is not well understood; probably, anti-EBV antibodies cross-react with self-antigens (i.e., I- and i- antigens expressed on erythrocytes membranes), due to molecular mimicry, triggering the activation of the complement cascade [2]. We conducted a review of the English literature, using PubMed, about pediatric cases of AIHA related to EBV infection; we revealed 16 cases (Table 1) [3–17]. Including our case, the median and mean age were 16 years and 12,7 years, respectively; 53% were female, while 35% were male. The main signs and symptoms were jaundice (76%), fever (65%), malaise and fatigue (41%), and hyperchromic urine (29%). The classical symptoms of infectious mononucleosis (i.e., exudative pharyngotonsillitis, lymphadenitis) were reported in just under half of the cases (41%); the diagnosis of EBV infection was made by serology in 82%. The majority of the episodes (76%) were cold AIHA, only one case was warm AIHA. The most common associated complication was hepatic injury (53%). All patients were hospitalized; although therapy with corticosteroids has generally poor efficacy in cold AIHA [18], steroids were administered in 70%; only 2 cases received also intravenous immunoglobulin (14%), and 2 patients needed plasma-exchange (14%) In one case, rituximab was also used (6%). The outcome was favorable in all patients, in whom it was reported. Our study has some limitation, including its retrospective design and missing data.

**Table 1:** EBV-related AIHA reported in literature and our case

Reference	Age/sex	Symptoms and signs	Mononucleosis symptoms	EBV diagnosis	Hb T/D bilirubin LDH Haptoglobin	Type of AIHA	Other complications	Therapy	Outcome
Jenkins et al. (1965)	18y/F	Fever Jaundice	No	Serology	Hb 3.8 g/dl T/D bilirubin 4.2/3.2 mg/dl LDH NR Haptoglobin NR	Cold	NR	NR	NR
Wilkinson et al. (1973)	16y/M	Malaise Fever Urticaria	Yes	Serology	Hb 9.6 g/dl T/D bilirubin 1.7/NR mg/dl LDH NR Haptoglobin undetectable	Cold CD3+	NR	Steroids	Recovered
Bowman et al. (1974)	18y/NR	Headache Myalgia Malaise Jaundice	Yes	Serology	Hb 6.9 g/dl T/D bilirubin 3.1/0.2 mg/dl LDH 2200 U/l Haptoglobin 0 mg/dl	Cold CD3+	NR	Steroids RBC transfusion	Recovered
Chambers et al. (1986)	17y/F	Jaundice Fever	No	Serology	Hb 9.4 g/dl T/D bilirubin 15.4/8.4 mg/dl LDH 472 U/l Haptoglobin NR	Cold	Acute hepatitis	Steroids	Recovered
Rollof et al. (1989)	18y/M	Jaundice Fever	No	Serology	Hb 5.6 g/dl T/D Bil 27.19/NR mg/dl LDH NR Haptoglobin NR	Cold	NR	Steroids RBC transfusion	Recovered
Palanduz et al. (2002)	7y/F	Diarrhea Fatigue Jaundice Hepatomegaly Splenomegaly	No	Serology	Hb 3.9 g/dl T/D Bil 14.8/13.8 mg/dl LDH 134.5 U/l Haptoglobin NR	Warm	Hepatic failure	Steroids	Recovered
Merino et al. (2006)	16y/M	Jaundice Stupor	Yes	Serology	Hb 4.9 g/dl T/D Bil NR LDH NR Haptoglobin NR	Cold	Erythrophagocytosis	Steroids Intravenous IgG Plasma-exchange Rituximab	Recovered
Place et al. (2007)	18y/F	Vomiting Jaundice	Yes	PCR EBV	Hb 7.1 g/dl T/D Bil 75.7/39.2 mg/dl LDH NR Haptoglobin NR	Cold	Cholestatic hepatitis	Steroids Plasma-exchange	Recovered
Mason et al. (2008)	13y/F	Abdominal pain Jaundice Dark urine Headache Malaise	No	Serology	Hb 7.7 g/dl T/D Bil 6.5/2.8 mg/dl LDH 1171 U/L Haptoglobin NR	Cold	Acute hepatitis	RBC transfusion	Recovered
Akin et al. (2011)	2y/M	Pallor Palpitation Fever Scleral icterus Hematuria	No	Serology	Hb 6.6 g/dl Indirect bilirubin 2.4 mg/dl LDH 2507 U/l Haptoglobin NR	CD3+ No cold agglutinin	NR	Steroids	Recovered
Akin et al. (2011)	2.5y/M	Pallor Palpitation Fever Scleral icterus Hematuria	No	Serology	Hb 3.9 g/dl Indirect bilirubin 3.5 mg/dl LDH 5800 U/l Haptoglobin NR	CD3+ No cold agglutinin	Acute renal failure	Steroids	Recovered
Ontanilla-Clavijo et al. (2017)	15y/F	Fever Headache	Yes	Serology PCR EBV	Hb 7.2 g/dl T/D Bil 14.8/13.8 mg/dl LDH NR Haptoglobin NR	Cold	Acute hepatitis	Prednisone Acyclovir	Recovered
Amer et al. (2018)	9 m/NR	Fever Splenomegaly Purpuric rash	Yes	Serology	Hb 5.7 g/dl T/D Bil NR LDH NR Haptoglobin NR	NR	Nephrotic syndrome Thrombocytopenia	RBC transfusion	Recovered
Mantadakis et al. (2018)	8y/F	Jaundice Fatigue Fever Dark urine	No	Serology PCR EBV	Hb 8.9 g/dl T/D Bil 6.9/4.3 mg/dl LDH 493 U/l Haptoglobin 8 mg/dl	Cold	Acute hepatitis	Steroids	Recovered
Dematapitiya et al. (2019)	18y/F	Malaise Fatigue Fever	Yes	Serology	Hb 8.6 g/dl T/D Bil 1.3/NR mg/dl LDH 1673 U/l Haptoglobin < 9 mg/dl	Cold	Hepatitis	Observation	Recovered
Teijido et al. (2019)	18y/M	Abdominal pain Nausea Vomiting Jaundice	No	Serology	Hb 11.1 g/dl T/D Bil 19.5/15.7 mg/dl LDH 1453 U/l Haptoglobin low	Cold	Hepatic dysfunction	Supportive treatment	Recovered
Our case	12y/F	Dark urine Jaundice	No	Serology	Hb 8,7 g/dl, T/D Bil 7.08/5.23 mg/dL LDH 1303 U/L Haptoglobin 7 mg/dl	Cold	NR	Steroid Intravenous IgG	Recovered

Abbreviations: y years, m months, M male, F female, NR not reported.

In conclusion, EBV-related AIHA is a rare condition, involving generally adolescent patients. Jaundice represents the main sign: it may depend on the association between hemolytic anemia and hepatic dysfunction, which is the most common complication, leading to elevated levels of both direct and indirect bilirubin. Although signs and symptoms of infectious mononucleosis may be absent, diagnostic test for acute EBV infection is always required, especially in case of hemolysis with cold agglutinin revealed by DAT.

## Data Availability

The datasets used and analyzed during the current study are available from the corresponding author on reasonable request.

## Abbreviations

EBV: Epstein-Barr virus
AIHA: Autoimmune hemolytic anemia
